# Supplementary material for: Optimization of MIMU Mounting Position on Shank in Posture Estimation Considering Muscle Protuberance
Source: Sensors (Basel). 2025 Apr 3;25(7):2273. doi: 10.3390/s25072273 (PMC11991399; doi:10.3390/s25072273)
Supplement: Supplementary file 1 [file sensors-25-02273-s001.zip › sensors-3542082-supplementary.pdf]

Table S1. Mean and standard deviation [°] of the maximum orientation change during Dorsiflexion for each subject. **(Supplementary data for Table 4)**

Dorsiflexion–relaxation

	Sagittal plane			Frontal plane			Horizontal plane		
	Lateral	Anterior	Medial tibia	Lateral	Anterior	Medial tibia	Lateral	Anterior	Medial tibia
**S1**	1.1	6.8	0.3	2.3	3.4	2.4	2.6	10.8	0.9
**S2**	0.9	4.9	0.5	2.4	3.5	1.4	2.2	11.2	1.7
**S3**	0.5	5.7	0.8	1.2	1.0	1.4	1.3	12.6	1.6
**S4**	0.7	2.7	0.3	1.9	1.8	1.2	0.9	3.0	0.8
**S5**	0.8	5.3	0.6	0.7	2.7	1.0	1.7	4.9	1.7
**S6**	1.3	8.0	0.6	1.1	1.9	1.3	2.5	7.5	1.0
**Average**	0.8	5.0	0.5	1.9	2.4	1.6	1.8	9.4	1.3
**SD**	0.3	1.8	0.2	0.7	1.0	0.5	0.7	3.8	0.4

Table S2. Mean and standard deviation [°] of the maximum orientation change during plantarflexion for each subject. **(Supplementary data for Table 4)**

Plantarflexion–relaxation

	Sagittal plane			Frontal plane			Horizontal plane		
	Lateral	Anterior	Medial tibia	Lateral	Anterior	Medial tibia	Lateral	Anterior	Medial tibia
**S1**	1.9	2.6	1.4	2.1	1.5	1.8	4.0	1.8	1.7
**S2**	1.4	1.8	0.6	1.5	0.4	1.3	4.2	1.6	1.4
**S3**	0.6	1.6	0.9	1.4	0.5	0.5	2.8	1.4	1.1
**S4**	0.7	1.9	2.0	2.7	1.3	0.8	1.7	2.2	0.6
**S5**	0.7	0.5	0.6	1.3	0.3	0.3	1.6	0.9	0.7
**S6**	1.1	2.2	1.4	0.4	0.3	0.2	1.3	0.5	1.0
**Average**	1.2	2.0	1.2	1.9	0.9	1.1	3.2	1.7	1.2
**SD**	0.5	0.7	0.6	0.8	0.5	0.6	1.3	0.6	0.4

Table S3. The mean RMSE and CC values [°] for each subject in slow gait.
(Supplementary data for Table 5, 6)

Slow

	RMSE									CC								
	Sagittal plane			Frontal plane			Horizontal plane			Sagittal plane			Frontal plane			Horizontal plane		
	Lateral	Anterior	Medial tibia	Lateral	Anterior	Medial tibia	Lateral	Anterior	Medial tibia	Lateral	Anterior	Medial tibia	Lateral	Anterior	Medial tibia	Lateral	Anterior	Medial tibia
**S1**	2.0	2.9	2.7	2.1	1.2	1.4	2.6	3.1	2.9	0.997	0.998	0.996	0.664	0.856	0.830	0.957	0.901	0.957
**S2**	1.5	1.6	1.9	2.7	1.2	1.0	3.7	2.1	2.1	0.998	0.999	0.998	0.738	0.924	0.957	0.920	0.933	0.933
**S3**	1.4	3.7	1.7	1.2	2.2	1.5	2.6	2.5	2.0	0.998	0.998	0.997	0.886	0.907	0.813	0.960	0.920	0.970
**S4**	1.4	3.0	2.2	2.0	3.3	2.1	2.6	1.8	3.5	0.998	0.997	0.997	0.895	0.713	0.845	0.976	0.980	0.977
**S5**	2.1	1.4	2.7	2.0	1.5	1.7	4.4	3.2	3.2	0.996	0.997	0.996	0.933	0.881	0.842	0.970	0.979	0.986
**S6**	1.9	2.1	2.6	1.6	2.8	1.4	5.1	4.7	4.5	0.996	0.997	0.996	0.907	0.400	0.891	0.935	0.969	0.968
**Average**	1.7	2.5	2.3	1.9	2.0	1.5	3.5	2.9	3.0	0.997	0.998	0.997	0.837	0.780	0.863	0.953	0.947	0.965
**SD**	0.31	0.90	0.42	0.52	0.89	0.34	1.08	1.02	0.95	0.001	0.001	0.001	0.109	0.201	0.053	0.022	0.034	0.018

Table S4. The mean RMSE and CC values [°] for each subject in medium-speed gait.
(Supplementary data for Table 5, 6)

Medium

	RMSE									CC								
	Sagittal plane			Frontal plane			Horizontal plane			Sagittal plane			Frontal plane			Horizontal plane		
	Lateral	Anterior	Medial tibia	Lateral	Anterior	Medial tibia	Lateral	Anterior	Medial tibia	Lateral	Anterior	Medial tibia	Lateral	Anterior	Medial tibia	Lateral	Anterior	Medial tibia
**S1**	2.6	4.3	3.2	3.2	1.9	2.1	3.2	2.8	2.8	0.998	0.998	0.997	0.580	0.764	0.627	0.956	0.935	0.946
**S2**	2.0	1.7	1.8	3.3	1.1	1.8	4.3	3.4	2.3	0.998	0.999	0.998	0.793	0.952	0.911	0.941	0.929	0.934
**S3**	1.9	4.2	2.1	1.5	2.4	1.4	2.3	2.3	2.7	0.996	0.996	0.996	0.871	0.882	0.783	0.965	0.962	0.973
**S4**	1.9	2.5	2.3	2.7	3.3	2.1	4.7	2.5	3.4	0.997	0.999	0.997	0.764	0.782	0.748	0.960	0.972	0.959
**S5**	2.6	2.1	3.6	2.5	2.4	2.8	4.2	5.4	5.1	0.998	0.998	0.997	0.952	0.898	0.865	0.970	0.974	0.983
**S6**	1.9	1.9	2.4	2.2	3.7	1.6	5.8	5.7	5.2	0.998	0.998	0.997	0.711	0.117	0.828	0.934	0.970	0.973
**Average**	2.2	2.8	2.6	2.5	2.5	2.0	4.1	3.7	3.6	0.997	0.998	0.997	0.779	0.732	0.794	0.955	0.957	0.961
**SD**	0.35	1.16	0.69	0.67	0.94	0.49	1.22	1.48	1.24	0.001	0.001	0.001	0.129	0.310	0.100	0.014	0.020	0.019

Table S5. The mean RMSE and CC values [°] for each subject in fast gait.

(Supplementary data for Table 5, 6)

Fast

	RMSE									CC								
	Sagittal plane			Frontal plane			Horizontal plane			Sagittal plane			Frontal plane			Horizontal plane		
	Lateral	Anterior	Medial tibia	Lateral	Anterior	Medial tibia	Lateral	Anterior	Medial tibia	Lateral	Anterior	Medial tibia	Lateral	Anterior	Medial tibia	Lateral	Anterior	Medial tibia
**S1**	2.3	2.8	2.8	2.2	2.1	2.1	3.3	3.5	2.9	0.997	0.998	0.997	0.803	0.816	0.769	0.975	0.973	0.979
**S2**	2.8	1.7	2.0	3.3	1.7	1.5	6.2	5.4	3.4	0.998	0.998	0.998	0.852	0.912	0.876	0.918	0.932	0.955
**S3**	2.0	3.8	2.1	2.0	2.3	1.9	5.8	6.0	3.5	0.996	0.998	0.997	0.664	0.890	0.630	0.955	0.955	0.971
**S4**	2.1	3.8	3.0	3.0	4.3	3.0	5.0	4.1	3.7	0.998	0.999	0.998	0.667	0.789	0.604	0.927	0.942	0.943
**S5**	3.0	2.8	2.9	2.6	2.2	2.0	3.1	3.4	3.3	0.996	0.997	0.996	0.925	0.887	0.900	0.958	0.959	0.964
**S6**	1.8	3.1	2.2	2.9	3.5	2.1	5.4	5.2	5.4	0.998	0.998	0.997	0.845	0.055	0.847	0.923	0.954	0.967
**Average**	2.3	3.0	2.5	2.7	2.7	2.1	4.8	4.6	3.7	0.997	0.998	0.997	0.793	0.725	0.771	0.943	0.953	0.963
**SD**	0.47	0.77	0.46	0.51	1.00	0.50	1.28	1.10	0.88	0.001	0.000	0.001	0.106	0.332	0.127	0.023	0.014	0.013

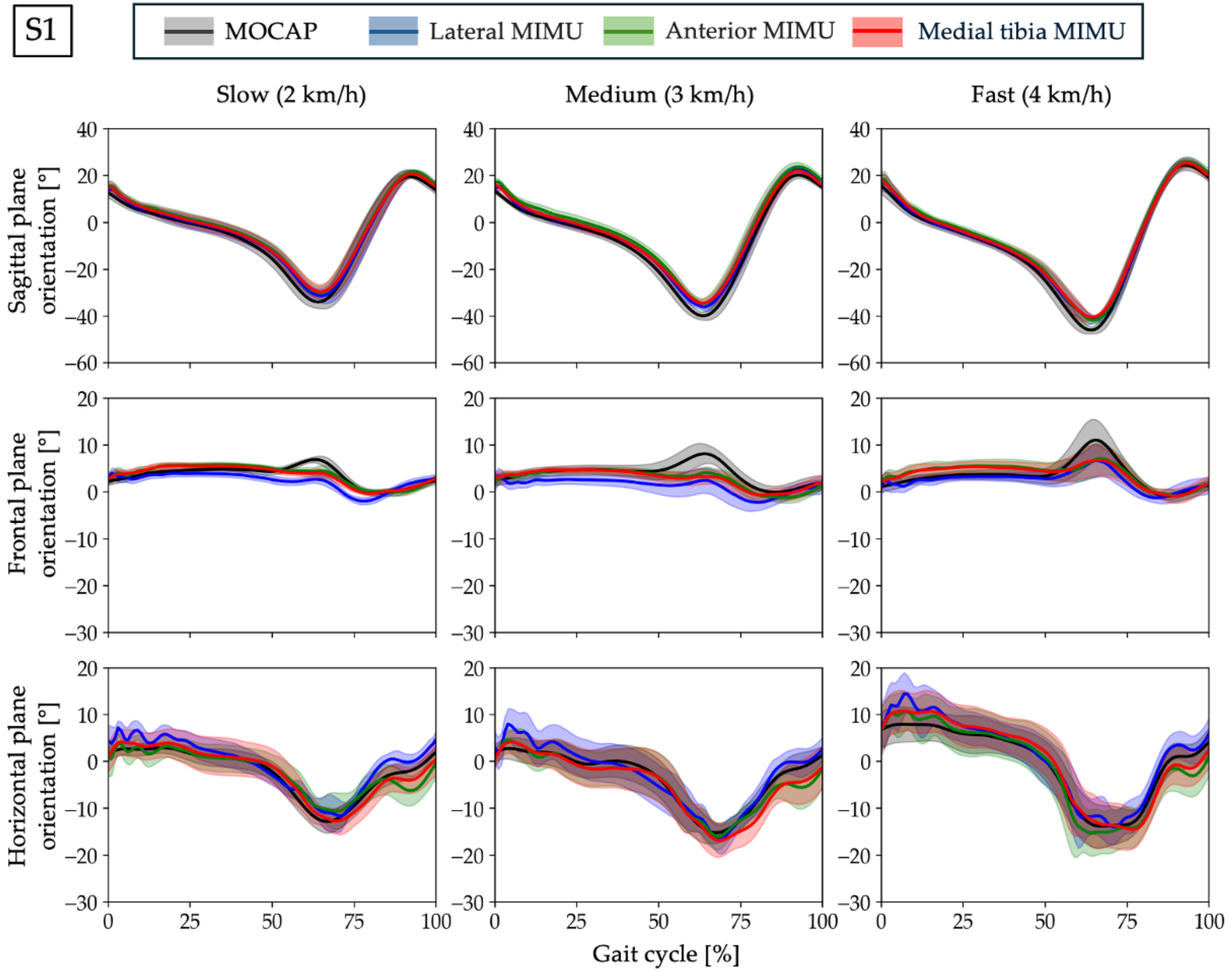

Figure S1. Waveform showing mean and standard deviation of shank posture data for subject 1
(Supplementary data for Figure 10)

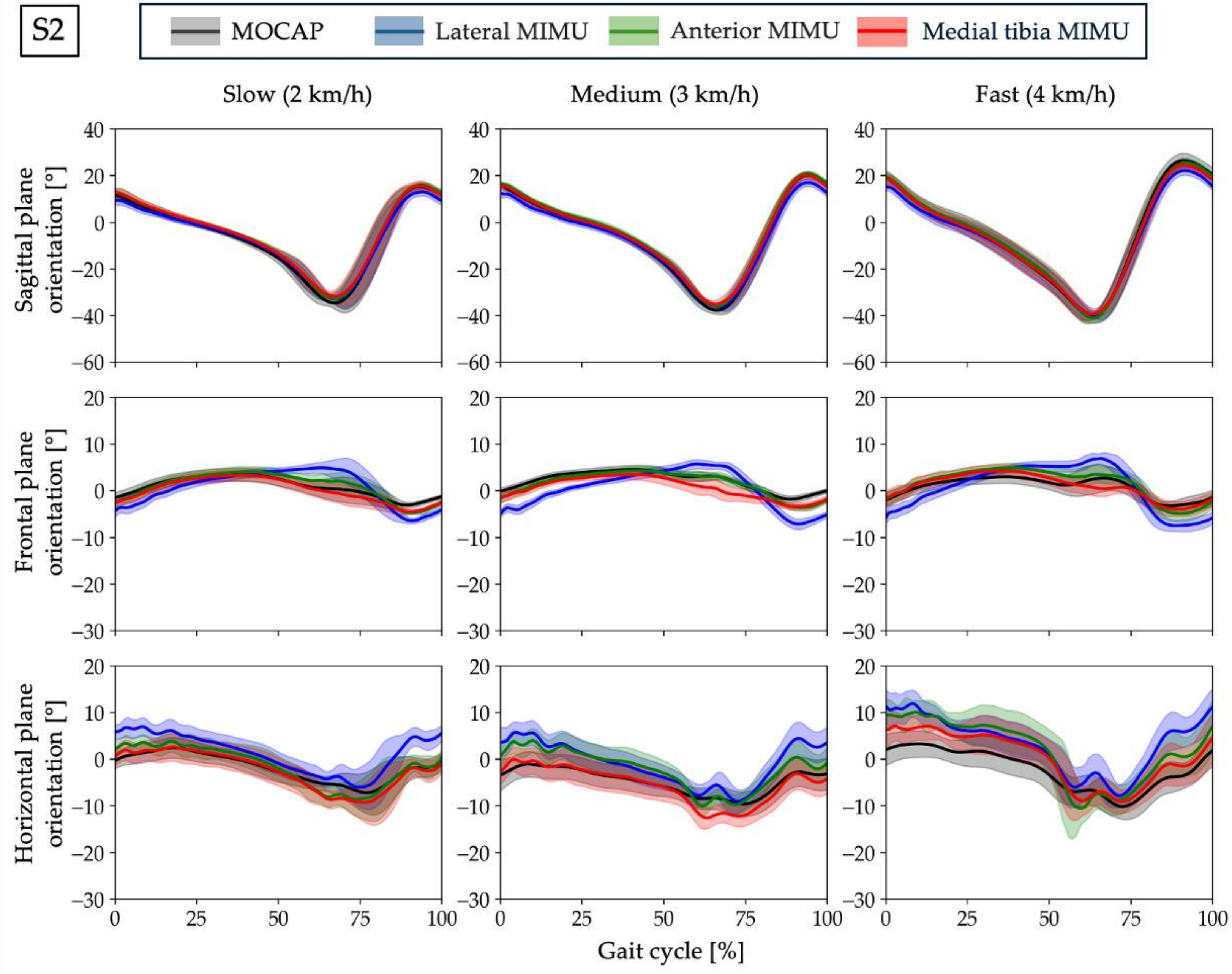

Figure S2. Waveform showing mean and standard deviation of shank posture data for subject 2

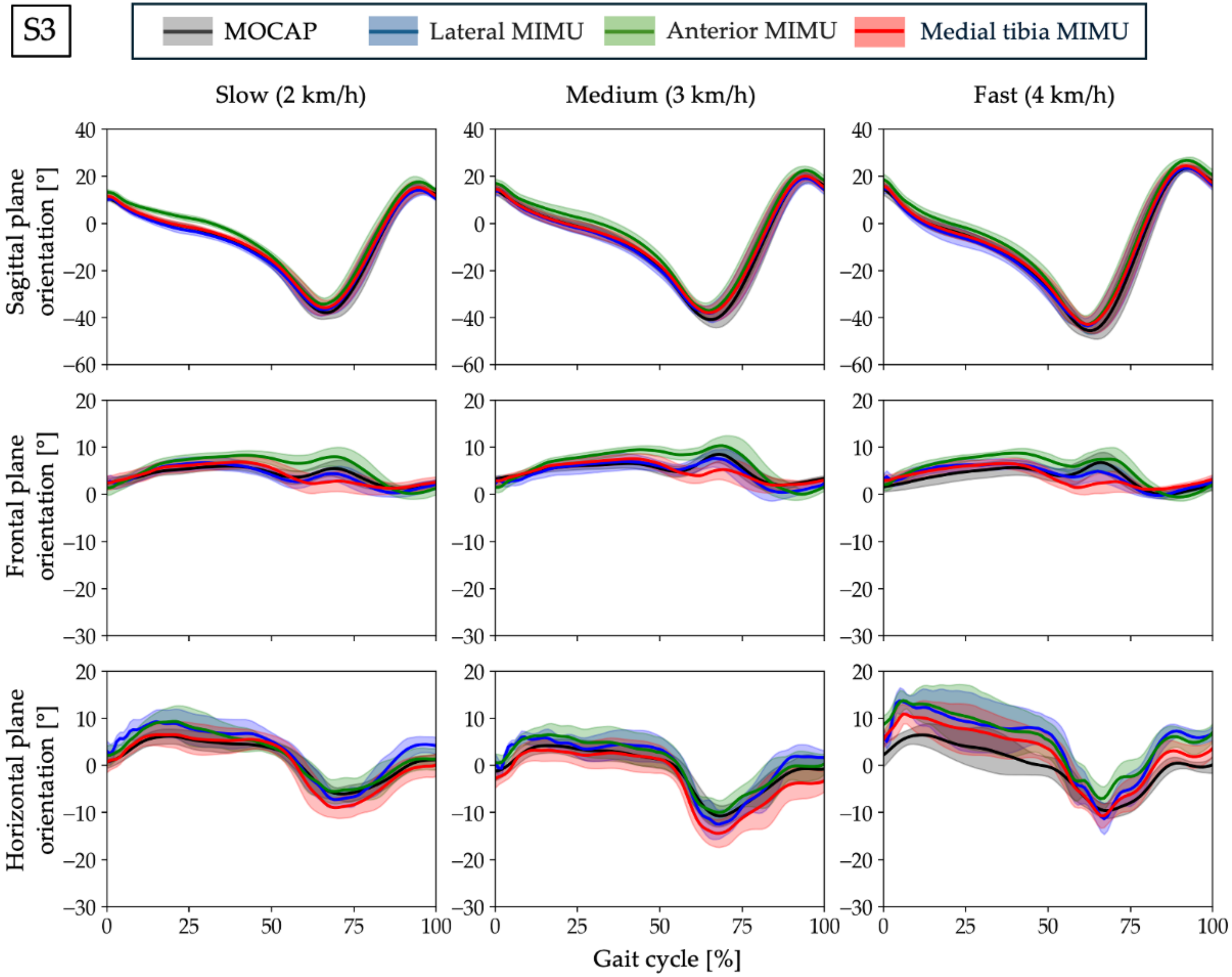

Figure S3. Waveform showing mean and standard deviation of shank posture data for subject 3

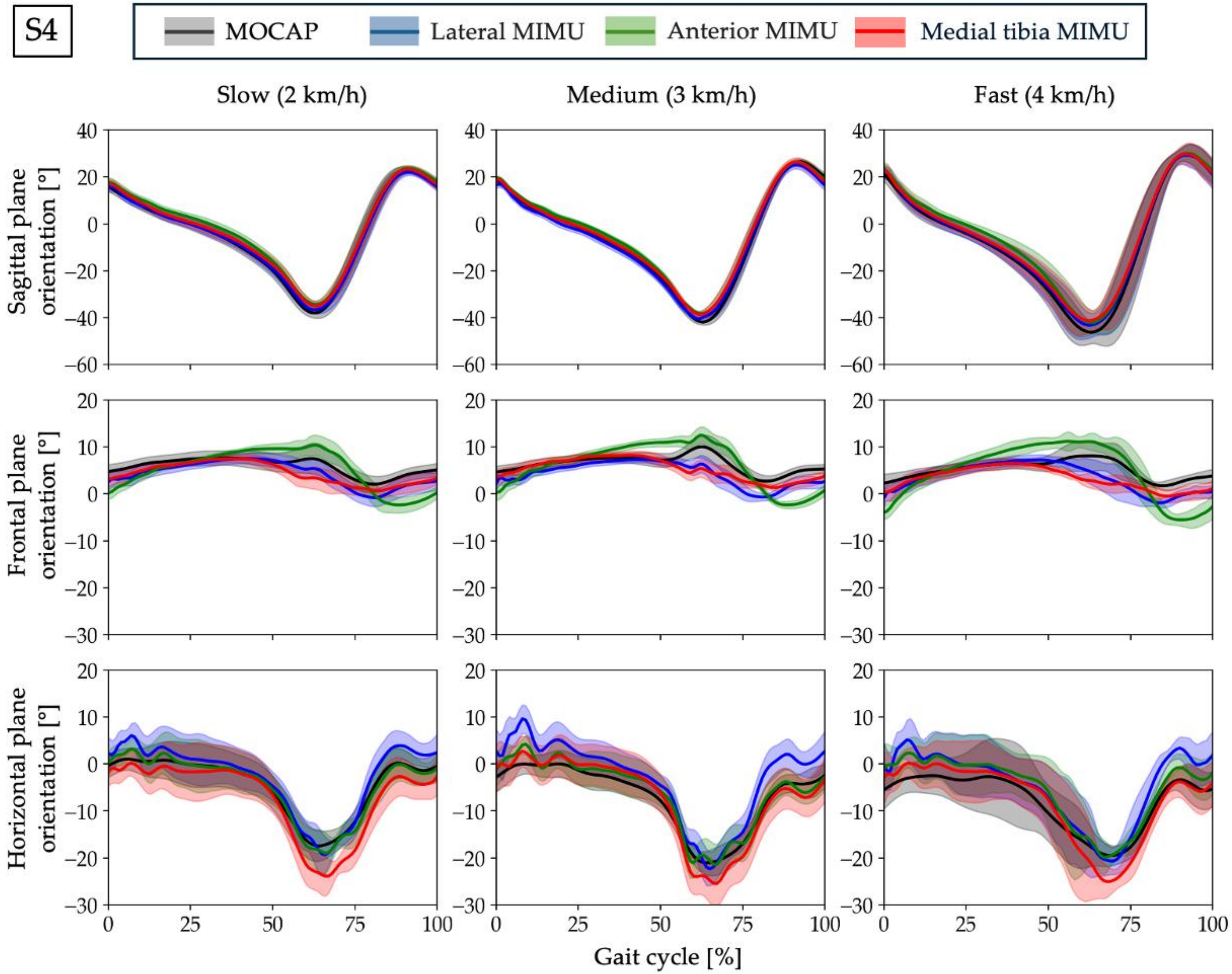

Figure S4. Waveform showing mean and standard deviation of shank posture data for subject 4

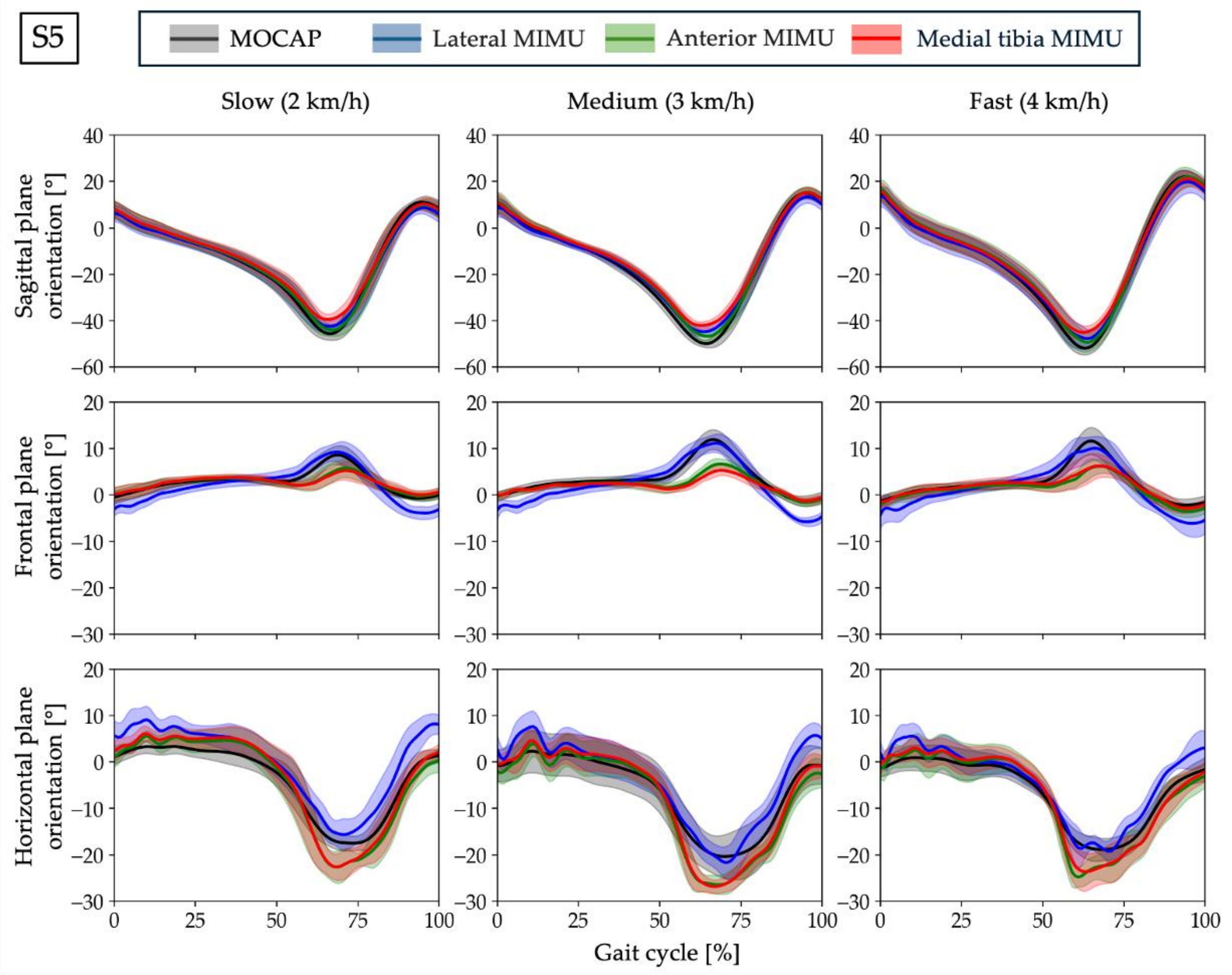

Figure S5. Waveform showing mean and standard deviation of shank posture data for subject 5

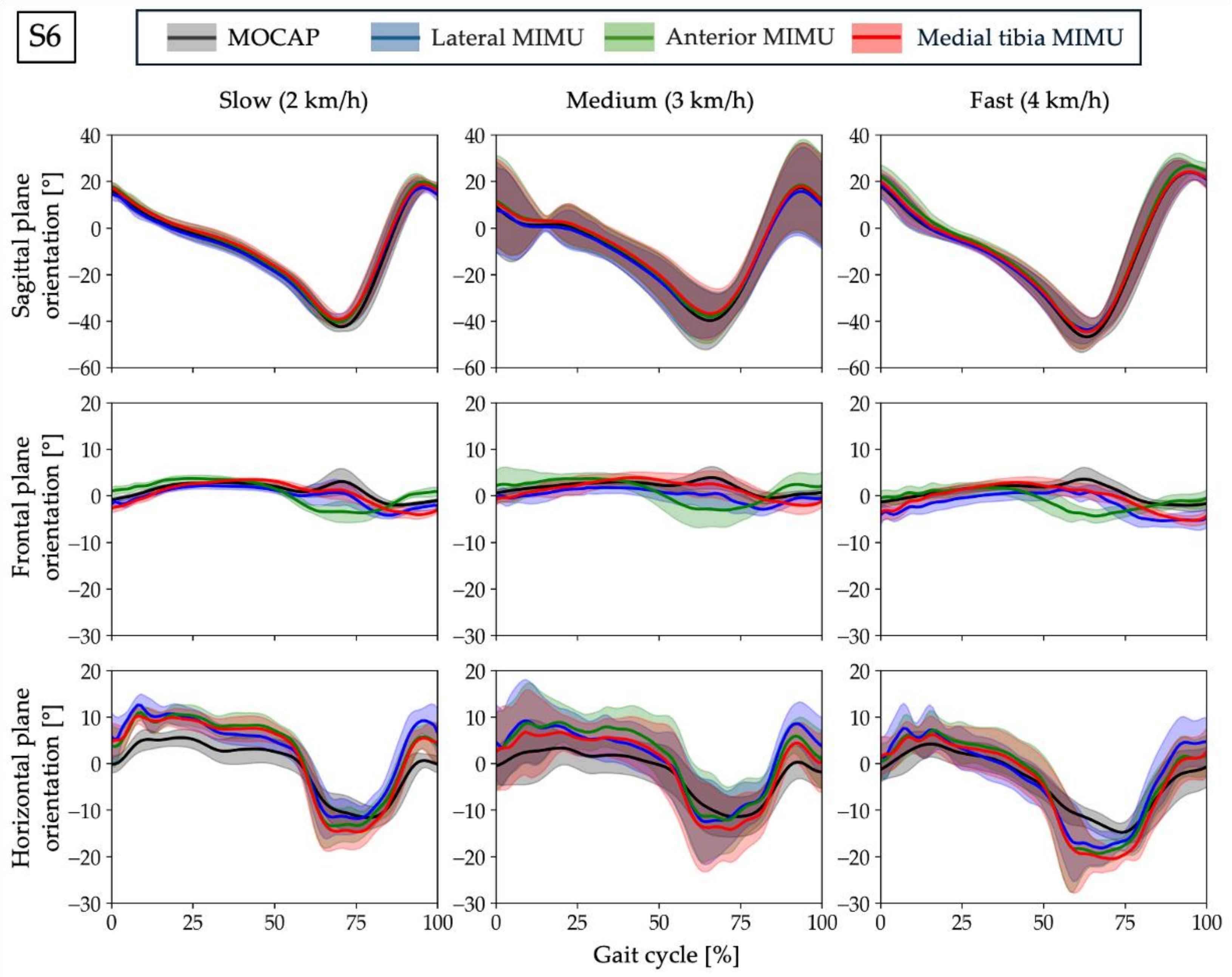

Figure S6. Waveform showing mean and standard deviation of shank posture data for subject 6
